# Spatial Linear Mixed Effects Modelling for OCT Images: SLME Model †

**DOI:** 10.3390/jimaging6060044

**Published:** 2020-06-05

**Authors:** Wenyue Zhu, Jae Yee Ku, Yalin Zheng, Paul C. Knox, Ruwanthi Kolamunnage-Dona, Gabriela Czanner

**Affiliations:** 1 Department of Eye and Vision Science, Institute of Life Course and Medical Sciences, University of Liverpool, a Member of Liverpool Health Partners, Liverpool L7 8TX, UK; jku@liverpool.ac.uk (J.Y.K.); yalin.zheng@liverpool.ac.uk (Y.Z.); pcknox@liverpool.ac.uk (P.C.K.); G.Czanner@ljmu.ac.uk (G.C.); 2 St Paul’s Eye Unit, Liverpool University Hospitals NHS Foundation Trust, a Member of Liverpool Health Partners, Liverpool L7 8XP, UK; 3 Department of Health Data Science, Institute of Population Health, University of Liverpool, a Member of Liverpool Health Partners, Liverpool L69 3GL, UK; kdrr@liverpool.ac.uk; 4 Department of Applied Mathematics, Liverpool John Moores University, Liverpool L3 3AF, UK

**Keywords:** spatial modelling, statistical analyses, correlated data, retinal imaging, optical coherence tomography, simulation, diabetic macular oedema

## Abstract

Much recent research focuses on how to make disease detection more accurate as well as “slimmer”, i.e., allowing analysis with smaller datasets. Explanatory models are a hot research topic because they explain how the data are generated. We propose a spatial explanatory modelling approach that combines Optical Coherence Tomography (OCT) retinal imaging data with clinical information. Our model consists of a spatial linear mixed effects inference framework, which innovatively models the spatial topography of key information via mixed effects and spatial error structures, thus effectively modelling the shape of the thickness map. We show that our spatial linear mixed effects (SLME) model outperforms traditional analysis-of-variance approaches in the analysis of Heidelberg OCT retinal thickness data from a prospective observational study, involving 300 participants with diabetes and 50 age-matched controls. Our SLME model has a higher power for detecting the difference between disease groups, and it shows where the shape of retinal thickness profiles differs between the eyes of participants with diabetes and the eyes of healthy controls. In simulated data, the SLME model demonstrates how incorporating spatial correlations can increase the accuracy of the statistical inferences. This model is crucial in the understanding of the progression of retinal thickness changes in diabetic maculopathy to aid clinicians for early planning of effective treatment. It can be extended to disease monitoring and prognosis in other diseases and with other imaging technologies.

## 1. Introduction

Diabetes is a major global health challenge. It affected approximately 463 million people (9.3% of the world’s population) in 2019, and this figure is estimated to rise to 700 million (10.9% of the world’s population) in 2045 [1]. Diabetic retinopathy (DR) is a common complication of diabetes, affecting approximately one-third of people with diabetes [2]. DR is the leading cause of visual loss in working age adults with visual loss caused by proliferative DR or Diabetic Macular Oedema (DMO). Where disease affects the central macula, a loss of central vision occurs with potentially severe quality of life impacts. In England in 2010, 7.12% (166325) of people with diabetes had DMO in one or both of their eyes, and 40% of DMO patients had clinically significant DMO with visual acuity poorer than 6/6 (Snellen) in at least one eye [3]. DMO is caused by an accumulation of fluid (oedema) in the macula thought to be secondary to vascular leakage. It involves retinal thickness changes in the macula. It has been identified that macular thickness is associated with visual loss [4,5]. OCT is now widely used for the diagnosis and monitoring of DMO as it is able to produce high-resolution cross-sectional images of the retina from which retinal thickness “maps” can be constructed [6]. A data-efficient method for analysis of spatial imaging data and the association between imaging data and clinical data are needed for more effective management of the disease.

**Figure 1:**
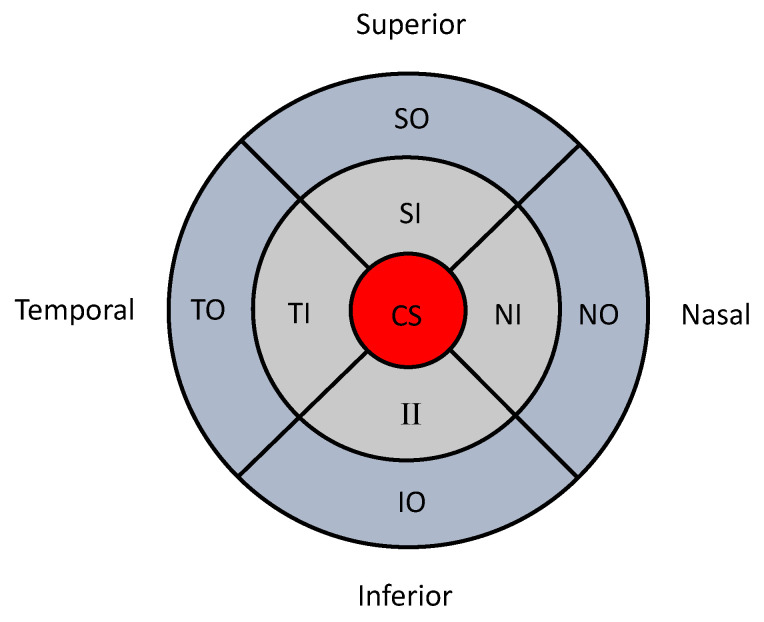
Early Treatment of Diabetic Retinopathy Study (ETDRS) grid centred on the fovea with the radii of the central circle being 500 μm, inner circle 1500 μm and outer circle 3000 μm.

Medical images are often divided into several clinically meaningful sectors to facilitate clinical investigations. The macula can be divided into nine subfields as initially described by the Early Treatment of Diabetic Retinopathy Study (ETDRS) research group [7]. These subfields comprise of three concentric circles with radii of 500, 1500 and 3000 μm subdivided into four regions (superior, temporal, inferior and nasal; Figure 1). These subfields are named by their location as the central subfield (CS), superior inner (SI), temporal inner (TI), nasal inner (NI), superior outer (SO), temporal outer (TO), inferior outer (IO) and nasal outer (NO). OCT measurements provide retinal thickness measurements for each of these nine subfields. Such spatial data measured in nine sectors are an example of lattice data in spatial statistics [8].

A key barrier to properly analyse such retinal imaging data is the limited understanding of the relationship between spatially collected data, i.e., spatial correlations. In some analyses only measurements of the central subfield (i.e., CS in Figure 1) are used, and the other measurements are disregarded. If the measurements of all sectors (i.e., all nine sectors in Figure 1) are considered in the analyses, there are two main statistical approaches used to analyse such imaging data. One is to ignore the spatial correlations (i.e., non-spatial approach), and the other is to consider the spatial correlations (i.e., spatial approach). For example, one non-spatial approach is to analyse data separately for each sector, which leads to multiple comparison problems. If the spatial dependency between the measurements of different sectors is not fully analysed, it will affect the precision of estimates, which may produce inaccurate results in statistical tests.

A spatial image analysis approach accounts for the spatial correlations when analysing the data by using spatial statistical models [8,9]. Therefore, a model is required which incorporates spatial information from measurements in all the subfields into the analysis. Such a model could provide valuable information for detecting retinal disease and discriminating between disease severity states [10]. Spatial statistical models have already been applied in other medical imaging contexts, such as functional neuroimaging and cardiac imaging, where spatial correlations are captured. For example, Bowman et al., constructed a spatial statistical model for cardiac imaging from single photon emission computed tomography [11]. They utilised a 20-sector model which considered the correlations among multiple perfusion measurements, in order to detect perfusion change in an individual’s left ventricle. Bernal-Rusiel et al. explored the spatial structures in Magnetic Resonance Image data in patients with Alzheimer’s disease [12]. Images were segmented into relatively small homogeneous regions, and a region-wise spatial model was developed. However, the application of spatial statistics to ophthalmic images has not yet been extensively studied. Moreover, the advantages of considering the spatial correlations are not fully understood. Hence the development of methods and further studies analysing spatial retinal imaging data are needed.

Another barrier relevant to the analysis of ophthalmic images is the issue of the unit of analysis. Often, the correlation between the two eyes from the same individual is ignored. Treating the two eyes as associated with each other can introduce spuriously small standard errors. Although there is continuing debate regarding this issue and methods are available for adjusting the correlation between the two eyes [13,14], the majority of studies do not take this problem into account when data from both eyes are available. This methodological barrier has not advanced much over the past two decades [15].

In this paper, we present a new statistical spatial inference framework for retinal images and study the effect of the spatial correlations on the analysis of spatial data. This framework is based on a linear nested mixed effects model with a spatial error structure (Gaussian, autoregressive-1, exponential and spherical) for the analysis of OCT imaging data, where correlations between eyes from the same patient and their individual clinical data are adjusted within the model. The model is estimated using restricted maximum likelihood estimation, which provides an unbiased estimation for both the fixed effects and the variance component for the mixed effects model. We compared the performance of our model with multivariate analyses of variance (MANOVA), which is one of the extensions of linear regression models called multivariate linear regression. In addition, we conducted a simulation study to validate our model and study the benefits of using a spatial modelling framework when different levels of spatial correlations exist. This paper is a substantial extension of previously published analysis [16]. In this paper, we refine the parameter estimation method, further validate the approach in a substantially larger clinical dataset, and extend comparisons to a three-group scenario.

The organization of the rest of the paper is as follows. The image dataset and the statistical modelling framework are presented in Section 2. In Section 3, we present results from the real data sets. Simulation setting and simulation results are presented in Section 4. Discussion of our work and the conclusions are presented in Section 5 and Section 6.

## 2. Methods

### 2.1. Dataset

The retinal imaging data used in this study are from a prospective observational clinical study (Early Detection of Diabetic Macular Oedema; EDDMO). All participants gave written, informed consent for inclusion before they participated in the EDDMO study, which was conducted in accordance with the principles laid down in the Declaration of Helsinki. Ethical approval was obtained from the UK’s Health Research Authority (North West - Preston Research Ethics Committee; REC reference 16/NW/0163; date of approval 31/3/2016). An interim, smaller dataset of 150 participants with diabetes who had been referred from the National Diabetic Eye Screening programme (NDESP) as screen positive to the Royal Liverpool University Hospital recruited at their first hospital visit were included in our analyses [17]. This dataset was used in our previous analysis [16] and will be used here for comparison with the full EDDMO dataset. Approximately 90% of these participants were Caucasians. Participants with diabetes who had co-existing pathologies (1 participant with intracranial lesions, and 4 participants with ocular pathologies) were excluded from the analysis). All participants were examined by an ophthalmologist with slit lamp biomicroscopy and had a dilated fundoscopy examination. All eyes were graded by an ophthalmologist for DR severity based on feature specific grading from the NDESP. Based on NDESP grading criteria, each eye of participants with diabetes was graded as having no evidence of maculopathy (M0) or having evidence of maculopathy (M1) [18]. Overall retinal thickness measurements for both the left and right eyes were obtained by Heidelberg Spectralis OCT. We excluded a small number of eyes that did not have OCT thickness data collected. A summary of the dataset stratified by clinical diagnosis based on slit lamp biomicroscopy is shown in Table 1. Although the measurements of both foveal centre point thickness and central subfield mean thickness are available using OCT, central subfield mean thickness is more commonly used in clinical research when tracking centre-involved DMO [19]. Therefore in the statistical analyses in this paper, we used central subfield mean thickness (CS) instead of foveal centre point thickness.

The full EDDMO dataset included data from 50 age-matched healthy controls and an additional 150 participants with diabetes. Self-reported ethnic background revealed that 96% of the healthy participants were Caucasian, and the characteristics of the additional 150 participants with diabetes was similar to the interim sample. Therefore in total, data from 300 participants with diabetes and 50 age-matched controls were available for inclusion. A summary of the full sample from EDDMO study is described in Table 2. We excluded the eyes that did not have OCT thickness data collected.

As for the clinical covariates, we included both patient-level demographic and clinical data (including age, gender, duration of diabetes, and smoking history), and eye-level clinical data (including axial length and best corrected distance visual acuity) in our model for three groups comparisons in this paper.

### 2.2. Statistical Model

We propose a spatial linear mixed effects (SLME) model for the spatially collected imaging data. It has the general form described in Equation (1), which is based on a linear mixed effects model with two levels of nested random effects.

In the SLME model, $Y_{ij}$ is the response vector for *i*th individual in the nested level *j* of grouping, $X_{ij}$ is the *p*-dimensional fixed effects vector (e.g., clinical information) associated with β, $b_{i}$ is the first level of random effects (e.g., individual level random effects) associated with $Z_{i}$, and $u_{ij}$ is the second level of random effects (e.g., eye level random effects nested within each individual) associated with $D_{ij}$.

(1) $$\begin{array}{cc} Y_{ij}= & X_{ij}\beta +Z_{i}b_{i}+D_{ij}u_{ij}+\epsilon_{ij},\;i=1,\ldots ,m;j=1,\ldots ,n_{i}\\ \; & b_{i}∼N\left(0,G_{1}\right),\;u_{ij}∼N\left(0,G_{2}\right),\;\epsilon_{ij}∼N\left(0,\Sigma_{s}\right) \end{array}$$

where the first level random effect $b_{i}$ is independent of the second level random effect $u_{ij}$, and $\epsilon_{ij}$ are within group error representing spatial correlations in the images which are assumed to be independent of random effects. The random effects $b_{i}$ and $u_{ij}$ are assumed to follow Gaussian distributions with variances $G_{1}$ and $G_{2}$ respectively. In the following sections, we explain the details of the SLME model and the parameter estimation.

### 2.3. Spatial Correlations in the SLME Model

The spatial correlations are used to describe the associations between the sectors of an image. These correlations are organised into a covariance matrix. The covariance matrix $\Sigma_{s}$ for $\epsilon_{ij}$ can be decomposed to $\Sigma_{s}=\sigma_{s}^{2}\Psi_{ij}$ where $\Psi_{ij}$ is a positive-definite matrix which can be decomposed to $\Psi_{ij}=\Lambda_{ij}C_{ij}\Lambda_{ij}$, and $\sigma_{s}$ is the parameter for residuals. $\Lambda_{ij}$ is a diagonal matrix and $C_{ij}$ is correlation matrix with parameter γ. In our model, $\Lambda_{ij}$ is a identity matrix and it is easy to write that $cor\left(\epsilon_{ijk},\epsilon_{ijk^{{}^{\prime }}}\right)={\left[C_{ij}\right]}_{k,k^{{}^{\prime }}}$, where $k,k^{{}^{\prime }}\left(k\neq k^{{}^{\prime }}\right)$ represent two different locations in image ij. There are a large number of correlations to be estimated if we consider the *k*, and $k^{{}^{\prime }}$ as associated, for example, 9×8/2 correlations for imaging data of 9 sectors. Such a large matrix can lead to a computationally unstable estimation and can cause matrix inversion problems.

A large number of parameters of the covariance matrix can be reduced via imposing a model of restriction. The spatial correlation $cor(\epsilon_{ijk},\epsilon_{ijk^{{}^{\prime }}})$ is modelled as either lag autoregressive model, Gaussian model, exponential model and spherical model where γ can take the value of $\gamma_{a}$, $\gamma_{g}$, $\gamma_{e}$, $\gamma_{s}$ respectively. For simplicity, let $s_{k,k^{{}^{\prime }}}$ denotes the spatial correlations between locations *k* and $k^{{}^{\prime }}$, and let $s_{k,k+1}$ denotes the spatial correlations between two neighbouring locations *k* and k+1 in one single image.

For the lag autoregressive model, the correlation function decreases in absolute value exponentially with lag δ(δ=1,2,…) model has the form of

(2) $$s_{k,k+1}=\gamma_{a}^{\delta }.$$

For spatial structured correlation, let $d_{k,k^{{}^{\prime }}}$ denotes the Euclidean distance between locations *k* and $k^{{}^{\prime }}$. The Gaussian correlation has the form of,

(3) $$s_{k,k^{{}^{\prime }}}=exp\left(-\gamma_{g}d_{k,k^{{}^{\prime }}}^{2}\right),$$

the exponential model has the form of

(4) $$s_{k,k^{{}^{\prime }}}=exp\left(-\gamma_{e}d_{k,k^{{}^{\prime }}}\right),$$

and the spherical model has the form of

(5) $$s_{k,k^{{}^{\prime }}}=1-1/2\left(3\gamma_{s}d_{k,k^{{}^{\prime }}}-\gamma_{s}^{3}d_{k,k^{{}^{\prime }}}^{3}\right).$$

### 2.4. Statistical Inference from the SLME Model

The idea is to derive the parameter estimates from the SLME model and then use the parameter estimates and their standard errors to make the inference. In this section, we explain the estimation of the parameters via a frequentist approach. We assume a multivariate normal distribution for $Y_{ij}$, and denote $Y_{ij}∼N_{n_{i}}\left(X_{ij}\beta ,H_{ij}\right)$ where $H_{ij}\equiv H_{ij}\left(\theta \right)$ is the covariance matrix for $Y_{ij}$ with parameter vector θ. For model (1), we aim to estimate the fixed effects (including β), make predictions for random effects (including $b_{i}$ and $u_{ij}$) and estimate the variance component (including θ). One of the most common methods is to use maximum likelihood (ML) estimation by maximizing the log-likelihood function, and it can be written as,

(6) $$l\left(\beta ,\theta \right)=-\frac{1}{2}\sum_{i=1}^{m}\log H_{ij}\left(\theta \right)-\frac{1}{2}\sum_{i=1}^{m}{\left(Y_{ij}-X_{ij}\beta \right)}^{{}^{\prime }}H_{ij}{\left(\theta \right)}^{-1}\left(Y_{ij}-X_{ij}\beta \right)+C$$

where *C* is a constant. By maximizing (6), we can obtain the ML estimates for β [20], and it can be shown that given θ,

(7) $$\hat{\beta }∼N_{p}\left(\beta ,{\left(X_{ij}^{{}^{\prime }}H_{ij}{\left(\theta \right)}^{-1}X_{ij}\right)}^{-1}\right)$$

However, ML estimation for the variance component θ will be biased downwards because the loss of degree of freedom in estimation for β. In contrast, we consider a restricted maximum likelihood (REML) estimation procedure to obtain less biased estimators for the variance components.

If we estimate the variance component θ via REML, then we can maximise the restricted log-likelihood function with respect to θ as follows,

(8) $$\begin{array}{cc} l_{R}\left(\theta \right)= & -\frac{1}{2}\sum_{i=1}^{m}\log H_{ij}\left(\theta \right)-\frac{1}{2}\sum_{i=1}^{m}\log X_{ij}^{{}^{{}^{\prime }}}H_{ij}{\left(\theta \right)}^{-1}X_{ij}\\ \; & -\frac{1}{2}\sum_{i=1}^{m}{\left(Y_{ij}-X_{ij}\hat{\beta }\right)}^{{}^{\prime }}H_{ij}{\left(\theta \right)}^{-1}\left(Y_{ij}-X_{ij}\hat{\beta }\right) \end{array}$$

apart from a constant, where

(9) $$\hat{\beta }={\left(\sum_{i=1}^{m}X_{ij}^{{}^{\prime }}H_{ij}{\left(\theta \right)}^{-1}X_{ij}\right)}^{-1}\left(\sum_{i=1}^{m}X_{ij}^{{}^{\prime }}H_{ij}{\left(\theta \right)}^{-1}Y_{ij}\right)$$

The REML estimator for θ can be obtained by numerical optimisation algorithms such as Newton-Raphson algorithm [21]. Once we have $\hat{\theta }$, we can insert it into (7) and the estimator $\hat{\beta }$ can be obtained. And the random effects $b_{i}$ and $u_{ij}$ can be predicted using their conditional expectations.

### 2.5. Parametrisation of the SLME for OCT Data

The SLME model can be flexibly parametrised to suit many applications. In our application to analyse the OCT retinal thickness data from EDDMO study, we used a nested linear random intercept model with spatial correlations, which is described as follows,

(10) $$\begin{array}{cc} y_{ij}= & x_{ij}\beta +b_{i}+u_{ij}+\epsilon_{ij},\;i=1,\ldots ,m;j=1,\ldots ,n_{i}\\ \; & b_{i}∼N\left(0,G_{1}\right),\;u_{ij}∼N\left(0,G_{2}\right),\;\epsilon_{ij}∼N\left(0,\sigma_{s}^{2}\Psi_{\mathrm{ij}}\right) \end{array}$$

where β is a parameter vector for the fixed effects, $b_{i}$ denotes the random effects for participant *i*, $u_{ij}$ denote the random effects for *j* eye in participant *i*, *m* is the number of participant and $\max n_{i}=2$. Let $x_{ijk}$ denotes the covariate for ith participant from j eye in sector k(k=1,…,9), and can be further partitioned as

(11) $$\begin{array}{cc} x_{ijk}\beta & =\beta_{0}+\beta_{1}\times x_{i}+\beta_{2}\times x_{ij}\\ \; & +\beta_{3}\times sector_{ijk}\\ \; & +\beta_{4}\times sector_{ijk}\times x_{i}+\beta_{5}\times sector_{ijk}\times x_{ij} \end{array}$$

where $x_{i}$ represent patient-level demographic or clinical data vector for *i*th participant; $x_{ij}$ is eye-level clinical data vector for *j* eye from *i*th participant, including clinical outcomes such as healthy eye without diabetes, diabetic eye without maculopahty and diabetic eye with maculopathy; $sector_{ijk}$ is a categorical variable from 1 to 9 which represent the 9 sectors in ETDRS grid with central subfield (CS) as a baseline; $sector_{ijk}\times x_{i}$ and $sector_{ijk}\times x_{ij}$ represent all possible interaction term between sector and patient-level clinical variables, and interaction term between sector and eye-level clinical variables, respectively.

The model (10) was fitted using the nlme-R package [22] and the spatial dependency $\Psi_{\mathrm{ij}}$ was fitted with structures as described in Section 2.3. Missing observations were tested whether they were missing at random and then handled using multiple imputation method in mice-R package [23].

### 2.6. Finding the Correct Parametrisation of the SLME

It is crucial to find a suitable parametrisation of the SLME model, i.e., model selection. There are two commonly used approaches for model selection, namely the Akaike information criterion (AIC) [24] and the Bayesian information criterion (BIC) [25], which are defined as,

(12) $$\mathrm{AIC}=-2l(\hat{\xi })+2p$$

(13) $$\mathrm{BIC}=-2l\left(\hat{\xi }\right)+2p\times \log\left(n\right)$$

where $\hat{\xi }$ is either ML or REML estimates of the parameter vector ξ from the model, *p* is the dimension of parameter vector ξ, *n* is the number of observations (but equals n−p when REML estimates are used), and l(·) is the log-likelihood function. The smaller the AIC or BIC is, the better the model is.

As for selecting the best mixed effects model in this paper, we used a top-down method to choose not only the optimal fixed effects but also the optimal random effects [26]. Firstly, we fit a saturated model with a simple covariance structure (e.g., a working independence structure), where all possible covariates and interaction terms are chosen as the fixed effects. Secondly, we investigate the optimal variance structure using AIC, BIC and liklihood ratio tests based on REML estimators. After selecting the optimal random effects structure, we then choose the optimal spatial structure. Finally, we refit the saturated model with the chosen covariance, and simplify the model by comparing the models with nested fixed effects using AIC, BIC and F-statistics based on ML estimation.

## 3. Results

We aimed to compare two models for spatial imaging profiles: a MANOVA model and our spatial model. MANOVA models do not utilise spatial correlations because they do not consider the relative spatial location of sectors. Our spatial model is built to explain the spatial imaging profiles, but it also utilised the spatial correlations.

To illustrate the proposed concepts, we used data from the EDDMO study. We conducted analysis on the interim dataset (150 participants with diabetes [16]), as well as the full dataset (300 participants with diabetes and 50 healthy age-matched controls). The demographics and clinical data of the participants included in the analysis are summarised as below (Table 3).

### 3.1. Statistical Spatial Modelling to Explain Whole Thickness Profiles: for Two Patients Groups

**Figure 2:**
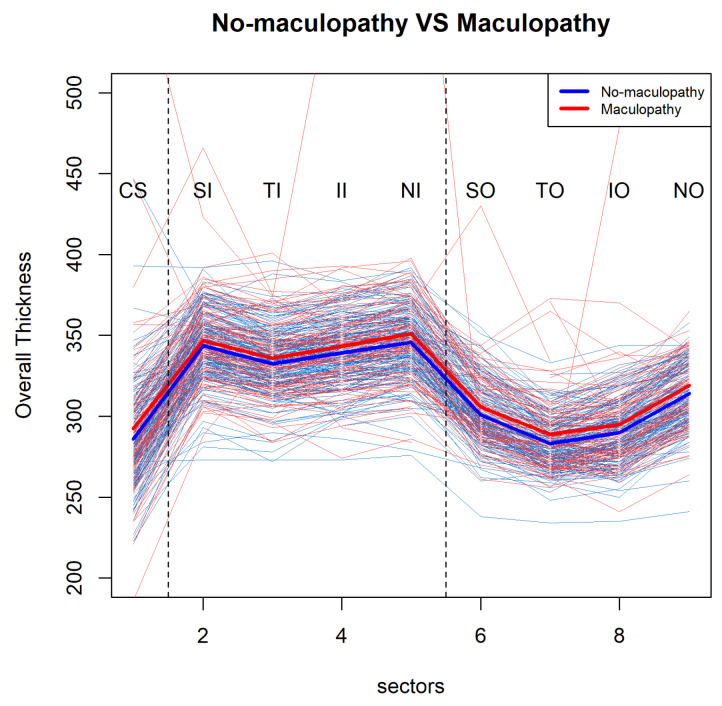
Pairwise comparisons for no-maculopathy and maculopathy eyes mean profiles of retinal overall thickness over 9 sectors (the smaller dataset).

Firstly, we illustrate the model concepts using the interim dataset with two patients groups. There are 150 participants with diabetes, and the data included in the analyses are summarised in Table 1. In total, data from 143 participants (i.e., 257 eyes) were used for the model selection, the parameter estimation and the inference. We made pairwise visualisations for mean profiles of retinal overall thickness over nine sectors at the participants’ baseline visit (Figure 2). This shows a large within group variability and it suggest a pattern for the mean profiles of retinal thickness over the nine sectors. We can see that the mean retinal thickness profile of the participants with diabetes with maculopathy (M1) is consistently higher than that of the participants with diabetes without maculopathy (M0), but this difference is quite subtle.

MANOVA was applied to study the two disease groups with respect to the OCT thickness data from nine locations of the ETDRS grid. The nine dependent variables were the OCT thickness from the nine locations, and the independent variable was the group, where the group is dichotomous with two levels: with maculopathy or without. The MANOVA did not find a statistical difference between the two disease groups in terms of retinal thickness (p=0.11>0.05). We also considered a Welch’s test with winsorized variances for retinal thickness between groups in CS (central subfield), which is one of the most important locations that a clinician will focus on in diagnosis. However we did not find any difference between retinal thickness in the group with maculopathy compared with the group without maculopathy in the CS (p=0.38>0.05). Then we considered the correlations between the two eyes and the spatial correlations between the nine sectors in statistical analyses using our model described in Section 2, which also allows heteroscedasticity between the groups. We investigated different spatial dependency structures described in Section 2.3; an exponential correlation structure was the most informative with the lowest AIC and BIC.

Our SLME model was applied to study the difference between two disease groups. With two levels of random effects model and an exponential correlation structure, we detected the difference in the main effect of diagnosis between the group with maculopathy and the group without maculopathy (p=0.02<0.05). The REML estimates of $\beta_{2}$ (i.e., the effect size between the groups) was 4.50 with standard error equal to 1.96. However, we did not detect a shape effect, which is measured as the interaction term between diagnosis and sector mathematically, between the group with maculopathy and the group without maculopathy (p=0.97). We also found a negative correlation between age and the mean retinal thickness profile (p<0.01). We further used a likelihood ratio test to confirm the significance of the eye within the patient random effects ($u_{ij}$) in the model (p<0.01). A detailed description of the model parameters can be found in the Supplementary Materials (Table S1).

In summary, we utilised spatial correlations and the whole imaging profile in the presented example above via the SLME model. It showed that the two disease groups are different (p=0.02). Although the MANOVA approach also explains the imaging profiles, it does not utilise the spatial correlations, and it did not find the difference between the two groups (p=0.11).

### 3.2. Statistical Spatial Modelling to Explain Whole Thickness Profiles: Full EDDMO Dataset with Three Participants Groups

**Figure 3:**
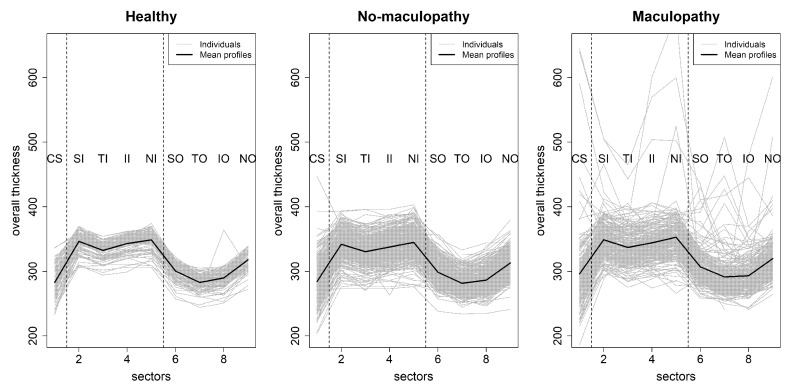
Visualizations of retinal overall thickness over nine sectors in the ETDRS grid for healthy group (the left panel), no-maculopathy group (the middle panel) and maculopathy group (the right panel). The grey lines represent profiles of all the individual eyes in this group, and the solid black lines represent the mean profiles for each group.

Next, we compare the analysis approaches to imaging data on the full EDDMO dataset (300 diabetic participants and 50 healthy participants). The data used in this section are summarised in Table 2. In total, data from 340 participants (i.e., 624 eyes) were used for the model selection, the parameter estimation and the statistical inference. Figure 3 shows the profiles of retinal overall thickness for healthy eyes, eyes with maculopthy and eyes without maculopathy in the nine ETDRS subfields. In this figure, we can see that the spatial profiles of the healthy eyes are similar, and it has a relative smaller variability in each of the nine locations. By contrast, the participants with diabetes have much larger within group variabilities. Moreover, some eyes in the maculopathy group have very high retinal thickness measurements in some specific sectors. These high retinal thickness measurements are a part of the usual range of retinal thickness in this patient cohort. Therefore, data from these participants are not treated as statistical outliers and is included for modelling purposes.

**Figure 4:**
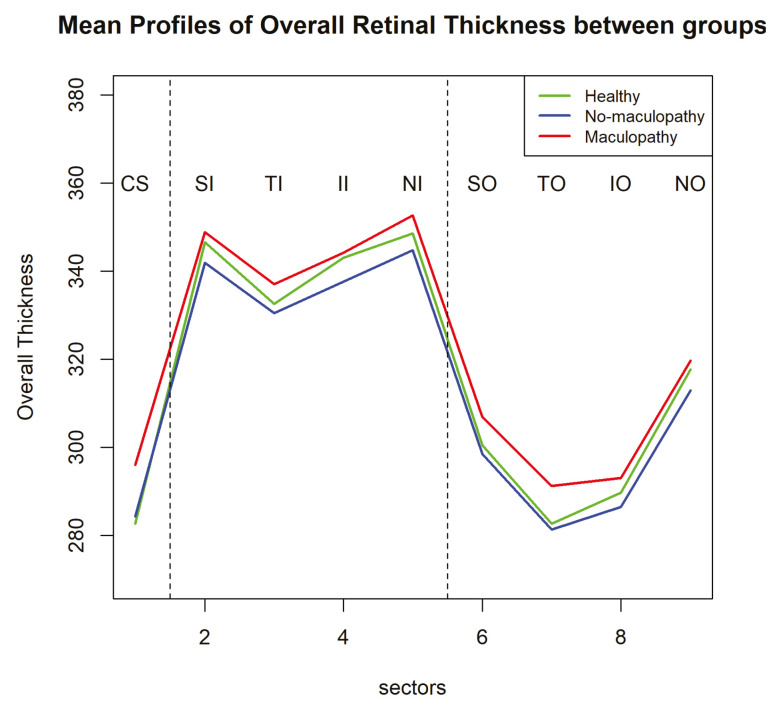
Visualizations for mean profiles of retinal overall thickness over the nine sectors in the ETDRS grid between the three groups (healthy, eyes with maculopathy and eyes without maculopathy). The green, blue, and red lines represent healthy eyes, eyes without maculopathy and eyes with maculopathy, respectively.

We plotted the mean retinal thickness profiles over nine sectors among the three groups in one figure (Figure 4) with an enlarged y-axis scale. The mean retinal thickness profile of the group with maculopathy is consistently higher than that of the group without maculopathy. In contrast, the difference between the healthy controls and all participants with diabetes (both with and without maculopathy) is very small. Interestingly, the profile of the healthy participants is in between of the profiles of the participants with diabetes without maculopathy and participants with diabetes with maculopathy.

Pairwise group comparisons using MANOVA was performed with respect to the OCT thickness data from nine locations of the ETDRS grid. It returned statistically significant results (all three *p* values are smaller than 0.01) between the three groups of participants (healthy, eyes with maculopathy, eyes without maculopathy) over the nine sectors. Using the fitted MANOVA model, we further assessed which of the nine locations showed significant differences across disease groups in terms of OCT measurements - in the pairwise group comparisons as a follow-up analysis of MANOVA. It shows that, for all sectors, the thickness was significantly different (p<0.01) between the eyes with maculopathy and the eyes without maculopathy. In contrast, eyes with maculopathy have thinner retinal thickness than healthy eyes only in SI and II sectors (both in imputed full data and in the original full data, *p* < 0.05). Pairwise comparison of eyes with maculopathy and healthy eyes also showed that the retinal thickness in the central subfield (p=0.017<0.05) and temporal outer subfield (p=0.016<0.05) is significantly thicker in the eyes with maculopathy.

Regarding our SLME model, we also found statistically significant difference among the three groups (p=0.0057<0.05). Our model confirms that there is a correlation between two eyes from the same patient (p<0.01), and the variability among the three groups are different (p<0.01). Moreover, an exponential correlation structure gives the most informative model, and is able to detect a shape effect, which is measured as the interaction term between diagnosis and sector mathematically. Using the model selection strategy for fixed effects described in Section 2.6, we also found that age is negatively correlated with the mean retinal thickness profiles (p<0.01). The REML estimates for age, variance component REML estimates for random effects and residuals, and heteroscedasticity range in the final selected model are summarised in Table 4. A more detailed description of all the model parameters can be found in the Supplementary Materials (Table S2).

We also used the SLME model to make pairwise comparisons among the three groups. As shown in Table 5, there is significant difference between eyes with maculopathy and eyes without maculopathy group in terms of the main effect of diagnosis, but there was no difference between participants with diabetes and healthy participants. However, there is a shape effect detected, which is measured as the interaction term between diagnosis and sector mathematically, between the eyes with maculopathy and the healthy participants (p<0.01), as well as between the eyes without maculopathy and healthy participants (p<0.01) (Table 6).

## 4. Simulation

In previous sections, we illustrated differences between MANOVA and our SMLE model in a real-world dataset. To give a further understanding of the performance of our spatial model, we carried out a simulation study to investigate the importance of incorporating spatial correlation in the statistical imaging analyses. We simplified the nested random intercept model (11) into a one-level random effects model with spatial exponential correlation. Covariates were chosen based on statistical analyses results from the EDDMO study, including nine locations from the ETDRS grid and a negatively continuous correlated risk factor (e.g., age). Only two disease groups (e.g., maculopathy group versus no-maculopathy group) were considered in the simulation as we are interested the effect of the spatial correlation and the power of our model rather than the clinical outcomes. The aim of our simulation study was to establish how well the spatial approach is able to estimate the risk factor and to test the difference between the diagnosis group in terms of the main effect and the shape effect.

In order to investigate how the spatial correlation can change the statistical inferences, we set three simulation scenarios in this section, one without correlation, one with a moderate ($\gamma_{e}=0.5$) and the other with a high correlation ($\gamma_{e}=0.1$) structure between different locations. Sample size were chosen as n=200 participants with one eye per individual where 70% of the eyes do not have maculopathy and 30% of the eyes have maculopathy. All the simulation results in this section are based on 1000 Monte Carlo replications. The simulation results including the true parameter values, sample size, Monte Carlo standard deviation, the mean of standard error estimates, the coverage probabilities for the estimates and the power to detect the shape effect are reported in Table 7, Table 8, Table 9 and Table 10. We compare results of our spatial model and a non-spatial model, which is a general linear model.

Firstly, we simulated a Scenario 1 with no correlation between the spatial thickness values. As expected, our SMLE model performed the same as the non-spatial model (Table 7) because there is no correlation between the spatial data. The parameter estimates were both practically unbiased, the Monte Carlo standard deviation agreed with the mean of standard error estimates, and the coverage probability was around 95%, which is reasonable.

Next, we simulated Scenarios 2 and 3, with moderate and high correlations between the spatial data, respectively. In Scenario 2, we used moderate exponential correlation with $\gamma_{e}=0.5$, and in Scenario 3, we used a high exponential correlation with $\gamma_{e}=0.1$ (Table 8 and Table 9 respectively). As shown in Table 8, there is a lower coverage probability in the non-spatial approach compared to our spatial approach. When higher spatial correlations exist, the coverage probability for the estimates of $\beta_{1}$ is much worse (Table 9). Using our spatial approach, the estimates of the parameters were practically unbiased with a reasonable coverage probability both in the moderate correlation setting and the high correlation setting. As expected, when there is no correlation between the simulated data as reported in Table 10, the spatial approach and the non-spatial approach were the same in detecting the shape effect (i.e., the interaction term). However, our spatial approach performed much better in the other two correlation settings.

## 5. Discussion

We used our SLME model to analyse the retinal thickness data in all nine subfields of the ETDRS grid from the EDDMO study. This approach is capable of incorporating spatial correlations in images, and investigating important information from subfields outwith CS in contrast to using CST only. It was also able to detect the association between disease and spatial topology that was undetected by a non-spatial approach. An exponential spatial correlation provides the best model with the lowest AIC and BIC. Using our spatial model, we found differences in mean retinal thickness between no-maculopathy versus maculopathy groups both in the interim dataset (150 participants) from our previous paper and the complete dataset (300 participants). However, MANOVA fails to detect the difference in the smaller dataset (150 participants). Moreover, no shape effect was detected between the retinal thickness of eyes with maculopathy and eyes without maculopathy. We also found that age is negatively correlated with the mean retinal thickness profile.

There are several advantages of our SLME model. From the clinical perspective, the principle advantage of our SLME model is the ability to pool the data from other sectors to effectively estimate the whole retinal thickness profile, thus increasing the power of between-group comparisons. Our method modelled the locations of the variables of interest in the images explicitly. It considers how far the location of the variable is from the centre, and the distance between the neighbouring variables of interest. Another advantage is that it works in the absence of some data, and does not require data imputation, but assumes that missing data are missing at random. Our model also incorporates clinical covariates into the image analyses, thus providing interpretable explanations of the results, which is important when studying disease etiology. Other advantages of our method include that it allows for heteroscedasticity between groups, and the correlation between two eyes from the same individual.

From the computational view, we provided further insight into the advantages of our SLME model using simulated data under different levels of spatial correlations in the images. Simulations demonstrated that our spatial approach is able to provide more accurate inference on the risk factor and has higher power to detect the main effect and the shape effect between diagnosis groups. It would be interesting to investigate the performance of our spatial approach over a wider range of sample sizes. Our spatial approach may perform better than a non-spatial approach with smaller sample sizes. However, when the sample size is large enough, the non-spatial approach may perform as well as our spatial approach in terms of detecting the main effect and the shape effect between groups.

In the clinical study (the EDDMO study), our SLME model did not find a statistically significant difference in the mean retinal thickness profile between healthy eyes and eyes with and without maculopathy. Nevertheless, Figure 4 shows that the spatial profile of healthy eyes is lower than the profile of maculopathy but slightly higher than that of the no-maculopathy group. Some researchers have reported a statistically significant decrease in retinal thickness when comparing diabetic patients without DMO to healthy participants, which may be explained by a loss of certain cell types in the retina in the early stages of preclinical DR [27,28,29]. In addition, we detected a shape difference between the retinal thickness profile of participants with diabetes and healthy participants. This suggests thickening of specific locations rather than general thickening across all locations in the early stages of DR and emphasises the potential importance of spatial relationships in the retina. This finding may be useful for the early detection of the DR and might help clinicians in planning early intervention.

The SMLE model is flexible and could be applied to investigate the spatial context or other features [30] of images from patients with other retinal diseases such as age related macular degeneration or retinal vascular occlusion. A further aim would be to develop flexible anisotropic spatial dependency structures adaptable to other medical images. It would also be useful to investigate the prediction of disease onset by extending our spatial modelling to spatio-temporal modelling by incorporating longitudinal datasets [31].

## 6. Conclusions

We have extended the standard analytic approach into spatial methods that adjust for spatial correlations and correlations between eyes from the same patients. In a clinical dataset, the SMLE model outperformed the non-spatial MANOVA by demonstrating higher power to detect differences. In simulated data, the SMLE model showed a higher power than non-spatial models. The increase was from 88.1% to 95.3%, 88.9% to 100% for moderate and high spatial correlations; hence the highest power increase was for circumstances where high correlations are present. In the future, the spatial approach could be extended into prediction or prognosis (i.e., predictive modelling) and the development of personal clinical management and monitoring tools. 

## Figures and Tables

**Table 1:** Number of eyes in the interim dataset used for the analysis of overall retinal thickness.

	M0	M1	Total
Left Eyes	91	40	131
Right Eyes	77	49	126
Total	168	89	257

**Table 2:** Number of eyes used for the analysis of overall retinal thickness in the full dataset from EDDMO study.

	Healthy	M0	M1	Total
Left Eyes	49	164	98	311
Right Eyes	47	154	112	313
Total	96	318	210	624

**Table 3:** Summary of the demographics and clinical data of the participants used for the analysis

	Interim Dataset		Full EDDMO Dataset		
	M0	M1	Healthy	M0	M1
Mean age in years ± SD (range)	54 ± 16 (20–86)	55 ± 15 (23–86)	55 ± 14 (22–85)	54 ± 15 (20–86)	53 ± 14 (23–86)
Gender (Female/Male)	37/52	17/37	24/26	67/103	48/72
Mean visual acuity in logMAR ± SD	0.04 ± 0.16	0.04 ± 0.23	−0.09 ± 0.10	0.05 ± 0.18	0.05 ± 0.19
Mean CST in μm ± SD	286 ± 30	293 ± 48	283 ± 24	284 ± 30	283 ± 24

SD, standard deviation; CST, central subfield thickness.

**Table 4:** Estimates for age, standard deviations of random effects, estimators for random effects and residuals, and heteroscedasticity range in the final model.

Some Model Parameters		Estimate (SD)	*p* Value
Age	${\hat{\beta }}_{1}$	−0.1984 (0.0727)	<0.01
Random effects	SD between individuals	18.3409	
	SD between one patient’s two eyes	1.4544	
Spatial correlations	${\hat{\gamma }}_{e}$; exponential correlation structure	1.4487	
Residuals	${\hat{\sigma }}_{s}$	23.796	
Heteroscedasticity scale among diagnossis group	Maculopathy	1	
	No-maculopathy	0.5369	
	Healthy	0.4588	

SD, standard deviation.

**Table 5:** Main effect for diagnosis using the SLME model

Main Effect	Group	Effect Size (SD)	*p* Value
Pairwise comparisons	Maculopathy vs No-maculopathy	3.4402 (1.1634)	<0.01
	Maculopathy vs Healthy	3.1873 (3.6003)	0.3726
	No-maculopathy vs Healthy	−3.2652 (2.5459)	0.1978

SD, standard deviation.

**Table 6:** Shape difference between diagnosis group using two-level of nested random effects spatial model with spatial correlation structure.

Shape Difference (i.e., Interaction Term: Diagnosis*Sector)	*p* Value
Overall comparison	<0.01
Maculopathy vs No-maculopathy	0.1346
Maculopathy vs Healthy	<0.01
No-maculopathy vs Healthy	<0.01

**Table 7:** Simulation studies: Scenario 1: no correlation between simulated spatial data (n=200).

Non-Spatial Approach Using Linear Regression			Spatial Approach Using SLME Model		
	Risk Factor ($\beta_{1}$)	Diagnosis for Main Effect ($\beta_{2}$)		Risk Factor ($\beta_{1}$)	Diagnosis for Main Effect ($\beta_{2}$)
True	−0.3	6.1	True	−0.3	6.1
Estimated	−0.2997	6.1013	Estimated	−0.2999	6.1015
SE	0.0012	0.1549	SE	0.0013	0.1548
SD	0.0013	0.1449	SD	0.0013	0.1448
CP	95.5%	96.5%	CP	98.5%	95.5%

SE, mean of standard error estimated; SD, Monte Carlo standard deviation of the estimated across the simulated data; CP, coverage probability for the estimated.

**Table 8:** Simulation studies: Scenario 2: moderate exponential spatial correlation between simulated spatial data ($\gamma_{e}$ = 0.5, n=200).

Non-Spatial Approach Using Linear Regression			Spatial Approach Using SLME Model		
	Risk Factor ($\beta_{1}$)	Diagnosis for Main Effect ($\beta_{2}$)		Risk Factor ($\beta_{1}$)	Diagnosis for Main Effect ($\beta_{2}$)
True	−0.3	6.1	True	−0.3	6.1
Estimated	−0.3001	6.0590	Estimated	−0.3000	6.0585
SE	0.0056	0.6927	SE	0.0083	0.6927
SD	0.0077	0.6563	SD	0.0077	0.6569
CP	84.0%	96.0%	CP	96.6%	95.9%

SE, mean of standard error estimates; SD, Monte Carlo standard deviation of the estimates across the simulated data; CP, coverage probability for the estimates.

**Table 9:** Simulation studies: Scenario 3: high exponential spatial correlation between simulated spatial data ($\gamma_{e}$ = 0.1, n=200)

Non-Spatial Approach Using Linear Regression			Spatial Approach Using SLME Model		
	Risk Factor ($\beta_{1}$)	Diagnosis for Main Effect ($\beta_{2}$)		Risk Factor ($\beta_{1}$)	Diagnosis for Main Effect ($\beta_{2}$)
True	−0.3	6.1	True	−0.3	6.1
Estimated	−0.3000	6.1180	Estimated	−0.3002	6.1120
SE	0.0056	0.6918	SE	0.0138	0.6927
SD	0.0170	0.7043	SD	0.0141	0.7033
CP	57.2%	95.1%	CP	94.9%	95.1%

SE, mean of standard error estimates; SD, Monte Carlo standard deviation of the estimates across the simulated data; CP, coverage probability for the estimates.

**Table 10:** Power of detecting the difference in shape between groups in spatial approach and non-spatial approach. The values in the table are the percentages of concluding the difference between disease groups in the Monte Carlo replications, if there is actually a difference, i.e., the power of the test.

The Level of Spatial Correlations	Non-Spatial Approach Using Linear Regression	Spatial Approach Using SLME Model
No correlation	100%	100%
Moderate correlation	88.1%	95.3%
High correlation	88.9%	100%

## Supplementary Materials

The following are available online at https://www.mdpi.com/2313-433X/6/6/44/s1, which includes two supplementary tables, i.e., Table S1: Detailed summary of all the estimated parameters in the final model in Section 3.1, and Table S2: Detailed summary of all the estimated parameters in the final model in Section 3.2.

## References

[1] Saeedi P., Petersohn I., Salpea P., Malanda B., Karuranga S., Unwin N., Colagiuri S., Guariguata L., Motala A.A., Ogurtsova K. (2019). Global and regional diabetes prevalence estimates for 2019 and projections for 2030 and 2045: Results from the International Diabetes Federation Diabetes Atlas. Diabetes Res. Clin. Pract..

[2] Yau J.W., Rogers S.L., Kawasaki R., Lamoureux E.L., Kowalski J.W., Bek T., Chen S.J., Dekker J.M., Fletcher A., Grauslund J. (2012). Global prevalence and major risk factors of diabetic retinopathy. Diabetes Care.

[3] Minassian D., Owens D.R., Reidy A. (2012). Prevalence of diabetic macular oedema and related health and social care resource use in England. Br. J. Ophthalmol..

[4] Catier A., Tadayoni R., Paques M., Erginay A., Haouchine B., Gaudric A., Massin P. (2005). Characterization of macular edema from various etiologies by optical coherence tomography. Am. J. Ophthalmol..

[5] Stunf Pukl S., Vidović Valentinčič N., Urbančič M., Irman Grčar I., Grčar R., Pfeifer V., Globočnik Petrovič M. (2017). Visual acuity, retinal sensitivity, and macular thickness changes in diabetic patients without diabetic retinopathy after cataract surgery. J. Diabetes Res..

[6] Hee M.R., Puliafito C.A., Wong C., Duker J.S., Reichel E., Rutledge B., Schuman J.S., Swanson E.A., Fujimoto J.G. (1995). Quantitative assessment of macular edema with optical coherence tomography. Arch. Ophthalmol..

[7] Early Treatment Diabetic Retinopathy Study Research Group (1991). Grading diabetic retinopathy from stereoscopic color fundus photographs—an extension of the modified Airlie House classification: ETDRS report No.10. Ophthalmology.

[8] Cressie N. (1992). Statistics for spatial data. Terra Nova.

[9] Lindquist M.A. (2008). The statistical analysis of fMRI data. Stat. Sci..

[10] MacCormick I.J., Williams B.M., Zheng Y., Li K., Al-Bander B., Czanner S., Cheeseman R., Willoughby C.E., Brown E.N., Spaeth G.L. (2019). Accurate, fast, data efficient and interpretable glaucoma diagnosis with automated spatial analysis of the whole cup to disc profile. PLoS ONE.

[11] Bowman F.D., Waller L.A. (2004). Modelling of cardiac imaging data with spatial correlation. Stat. Med..

[12] Bernal-Rusiel J.L., Reuter M., Greve D.N., Fischl B., Sabuncu M.R., Initiative A.D.N. (2013). Spatiotemporal linear mixed effects modeling for the mass-univariate analysis of longitudinal neuroimage data. Neuroimage.

[13] Ying G.s., Maguire M.G., Glynn R., Rosner B. (2017). Tutorial on biostatistics: Linear regression analysis of continuous correlated eye data. Ophthalmic Epidemiol..

[14] Ying G.s., Maguire M.G., Glynn R., Rosner B. (2018). Tutorial on Biostatistics: Statistical Analysis for Correlated Binary Eye Data. Ophthalmic Epidemiol..

[15] Zhang H.G., Ying G.S. (2018). Statistical approaches in published ophthalmic clinical science papers: A comparison to statistical practice two decades ago. Br. J. Ophthalmol..

[16] Zhu W., Ku J.Y., Zheng Y., Knox P., Harding S.P., Kolamunnage-Dona R., Czanner G. (2019). Spatial Modelling of Retinal Thickness in Images from Patients with Diabetic Macular Oedema. Annual Conference on Medical Image Understanding and Analysis.

[17] Public Health England Diabetic Eye Screening: Retinal Image Grading Criteria. https://www.gov.uk/government/publications/diabetic-eye-screening-retinal-image-grading-criteria.

[18] Public Health England (2012). Diabetic Eye Screening Feature Based Grading Forms. https://assets.publishing.service.gov.uk/government/uploads/system/uploads/attachment_data/file/582710/Grading_definitions_for_referrable_disease_2017_new_110117.pdf.

[19] BuAbbud J.C., Al-latayfeh M.M., Sun J.K. (2010). Optical coherence tomography imaging for diabetic retinopathy and macular edema. Curr. Diabetes Rep..

[20] Laird N.M., Ware J.H. (1982). Random-effects models for longitudinal data. Biometrics.

[21] Lindstrom M.J., Bates D.M. (1988). Newton—Raphson and EM algorithms for linear mixed-effects models for repeated-measures data. J. Am. Stat. Assoc..

[22] Pinheiro J., Bates D., DebRoy S., Sarkar D., R Core Team nlme: Linear and Nonlinear Mixed Effects Models.

[23] Van Buuren S., Groothuis-Oudshoorn K. (2011). mice: Multivariate Imputation by Chained Equations in R. J. Stat. Softw..

[24] Sakamoto Y., Ishiguro M., Kitagawa G. (1986). Akaike information criterion statistics.

[25] Schwarz G. (1978). Estimating the dimension of a model. Ann. Stat..

[26] Diggle P., Diggle P.J., Heagerty P., Liang K.Y., Heagerty P.J., Zeger S. (2002). Analysis of Longitudinal Data.

[27] Biallosterski C., Van Velthoven M.E., Michels R.P., Schlingemann R.O., DeVries J.H., Verbraak F.D. (2007). Decreased optical coherence tomography-measured pericentral retinal thickness in patients with diabetes mellitus type 1 with minimal diabetic retinopathy. Br. J. Ophthalmol..

[28] Netto M.P., Lima V.C., Pacheco M.A., Unonius N., Gracitelli C.P., Prata T.S. (2018). Macular Inner Retinal Layer Thinning in Diabetic Patients without Retinopathy Measured by Spectral Domain Optical Coherence Tomography. Med Hypothesis, Discov. Innov. Ophthalmol..

[29] Khan B., Quraishy M.M., Shams A. (2019). Central Macular Thickness: A Comparative Study of Diabetics Vs Healthy. Pak. J. Ophthalmol..

[30] MacCormick I.J., Zheng Y., Czanner S., Zhao Y., Diggle P.J., Harding S.P., Czanner G. (2017). Spatial statistical modelling of capillary non-perfusion in the retina. Sci. Rep..

[31] Vogl W.D., Waldstein S.M., Gerendas B.S., Schmidt-Erfurth U., Langs G. (2017). Predicting macular edema recurrence from spatio-temporal signatures in optical coherence tomography images. IEEE Trans. Med. Imaging.

