# Supplementary material for: Spatial Linear Mixed Effects Modelling for OCT Images: SLME Model
Source: J Imaging. 2020 Jun 5;6(6):44. doi: 10.3390/jimaging6060044 (PMC8321139; doi:10.3390/jimaging6060044)
Supplement: Supplementary file 1 [file jimaging-06-00044-s001.pdf]

Table S1: Detailed summary of all the estimated parameters in the final model in section 3.1

<table>
<tr><td colspan="3">Model parameters</td><td>Baseline</td><td>Estimate (SD)</td><td>p value</td></tr>
<tr><td rowspan="11">Fixed Effects</td><td colspan="3">Intercept</td><td>300.0442 (5.4680)</td><td>0.0000</td></tr>
<tr><td rowspan="8">Indicator variable for spatial locations</td><td>SI</td><td rowspan="8">CS</td><td>56.9670 (1.0986)</td><td>0.0000</td></tr>
<tr><td>TI</td><td>45.8307 (1.0986)</td><td>0.0000</td></tr>
<tr><td>II</td><td>52.7216 (1.0986)</td><td>0.0000</td></tr>
<tr><td>NI</td><td>59.5254 (1.0986)</td><td>0.0000</td></tr>
<tr><td>SO</td><td>14.9707 (1.3451)</td><td>0.0000</td></tr>
<tr><td>TO</td><td>-3.0782 (1.2823)</td><td>0.0165</td></tr>
<tr><td>IO</td><td>3.4038 (1.3136)</td><td>0.0096</td></tr>
<tr><td>NO</td><td>27.7981 (1.2823)</td><td>0.0000</td></tr>
<tr><td>Diagnosis</td><td>M1</td><td>M0</td><td>4.4969 (1.9584)</td><td>0.0235</td></tr>
<tr><td colspan="3">Age</td><td>-0.2678 (0.0965)</td><td>0.0063</td></tr>
<tr><td rowspan="2">Random effects</td><td colspan="3">SD between individuals</td><td colspan="2">16.0664</td></tr>
<tr><td colspan="3">SD between one individual’s two eyes</td><td colspan="2">7.9863</td></tr>
<tr><td>Spatial range</td><td colspan="3">Exponential correlation structure</td><td colspan="2">1.5081</td></tr>
<tr><td>Residuals</td><td colspan="3">SD</td><td colspan="2">22.5997</td></tr>
<tr><td rowspan="2">Heteroscedasticity scale among diagnosis group</td><td colspan="3">M1</td><td colspan="2">1.0000</td></tr>
<tr><td colspan="3">M0</td><td colspan="2">0.5792</td></tr>
</table>

Note that in this table, CS, SI, TI, II, NI, SO, TO, IO, NO represent the nine subfields in ETDRS grid (figure 1); M1, M0 represent maculopathy and no-maculopathy group, respectively; SD represents standard deviation

Table S2: Detailed summary of all the estimated parameters in the final model in section 3.2

| Model parameters | | | Baseline | Estimate (SD) | p value |
|---|---|---|---|---|---|
| Fixed Effects | Intercept | | | 295.7871 (4.1325) | 0.0000 |
| | Indicator variable for spatial locations | SI | CS | 57.6132 (0.8545) | 0.0000 |
| | | TI | | 46.176 (0.8545) | 0.0000 |
| | | II | | 53.3176 (0.8545) | 0.0000 |
| | | NI | | 60.4843 (0.8545) | 0.0000 |
| | | SO | | 14.8498 (1.0171) | 0.0000 |
| | | TO | | -2.9623 (0.9902) | 0.0028 |
| | | IO | | 2.0268 (1.0041) | 0.0436 |
| | | NO | | 28.8265 (0.9919) | 0.0000 |
| | Diagnosis | M1 | M0 | 7.3989 (1.9293) | 0.0002 |
| | | MH | | -2.4351 (3.1261) | 0.4366 |
| | Age | | | -0.1984 (0.0727) | 0.0067 |
| | Interaction term between spatial locations and diagnosis group | SI : M1 | CS : M0 | -4.7799 (2.1369) | 0.0253 |
| | | TI : M1 | | -5.1856 (2.1369) | 0.0153 |
| | | II : M1 | | -5.1748 (2.1369) | 0.0155 |
| | | NI : M1 | | -3.8509 (2.1369) | 0.0716 |
| | | SO : M1 | | -4.4757 (2.5294) | 0.0769 |
| | | TO : M1 | | -1.7996 (2.4763) | 0.4674 |
| | | IO : M1 | | -4.5821 (2.5092) | 0.0679 |
| | | NO : M1 | | -5.1170 (2.4770) | 0.0389 |
| | | SI : MH | | 6.2401 (1.5800) | 0.0001 |
| | | TI : MH | | 3.6781 (1.5800) | 0.0200 |
| | | II : MH | | 7.0782 (1.5800) | 0.0000 |
| | | NI : MH | | 5.4324 (1.5800) | 0.0006 |
| | | SO : MH | | 2.9523 (1.8456) | 0.1097 |
| | | TO : MH | | 2.9519 (1.8310) | 0.1070 |
| | | IO : MH | | 4.9837 (1.8385) | 0.0067 |
| | | NO : MH | | 6.1839 (1.8319) | 0.0007 |
| Random effects | SD between individuals | | | 18.3409 | |
| | SD between one individual’s two eyes | | | 1.4544 | |
| Spatial range | Exponential correlation structure | | | 1.4487 | |
| Residuals | SD | | | 23.7962 | |
| Heteroscedasticity scale among diagnosis group | M1 | | | 1.0000 | |
| | M0 | | | 0.5369 | |
| | MH | | | 0.4588 | |

Note that in this table, CS, SI, TI, II, NI, SO, TO, IO, NO represent the nine subfields in ETDRS grid (figure 1); M1, M0, MH represent maculopathy, no-maculopathy and healthy group, respectively; SD represents standard deviation.
